# Investigating young women’s motivations to engage in early mammography screening in Switzerland: results of a cross-sectional study

**DOI:** 10.1186/s12885-017-3180-1

**Published:** 2017-03-21

**Authors:** Nanon H. M. Labrie, Ramona Ludolph, Peter J. Schulz

**Affiliations:** 1 Department of Medical Psychology, Academic Medical Center, University of Amsterdam, Meibergdreef 9, 1100 DD Amsterdam, The Netherlands; 2 Institute of Communication & Health, Faculty of Communication Sciences, Università della Svizzera italiana, USI, Via G. Buffi 13, CH-6904 Lugano, Switzerland

**Keywords:** Breast cancer, Mammography screening, Knowledge, Fear, Risk perceptions, Ego-involvement, Switzerland, Women aged 30-49

## Abstract

**Background:**

The scientific and public debate concerning organized mammography screening is unprecedentedly strong. With research evidence concerning its efficacy being ambiguous, the recommendations pertaining to the age-thresholds for program inclusion vary between – and even within – countries. Data shows that young women who are not yet eligible for systematic screening, have opportunistic mammograms relatively often and, moreover, want to be included in organized programs. Yet, to date, little is known about the precise motivations underlying young women’s desire and intentions to go for, not medically indicated, mammographic screening.

**Methods:**

A cross-sectional online survey was carried out among women aged 30-49 years (*n* = 918) from Switzerland.

**Results:**

The findings show that high fear (*β* = .08, *p* ≤ .05), perceived susceptibility (*β* = .10, *p* ≤ .05), and ego-involvement (*β* = .34, *p* ≤ .001) are the main predictors of screening intentions among women who are not yet eligible for the systematic program. Also, geographical location (Swiss-French group: *β* = .15, *p* ≤ .001; Swiss-Italian group: *β* = .26, *p* ≤ .001) and age (*β* = .11, *p* ≤ .001) play a role. In turn, breast cancer knowledge, risk perceptions, and educational status do not have a significant impact.

**Conclusions:**

Young women seem to differ inherently from those who are already eligible for systematic screening in terms of the factors underlying their intentions to engage in mammographic screening. Thus, when striving to promote adherence to systematic screening guidelines – whether based on unequivocal scientific evidence or policy decisions – and to allow women to make evidence-based, informed decisions about mammography, differential strategies are needed to reach different age-groups.

## Background

Since the publication of the findings of the 25-year Canadian National Breast Screening Study (CNBSS) in February 2014, the scientific and public debate concerning organized mammography screening has flared up. The results of the CNBSS suggest that annual screening of women aged 40 to 59 years does not reduce breast cancer mortality beyond that of physical examination or usual care and that, as a result, policy makers should urgently reassess the rationale for mammography screening [1]. While the CNBSS has received considerable methodological criticism [2–5], a recent evaluation report issued by the International Agency for Research on Cancer (IARC) draws similar conclusions. The IARC work group evaluates that while the “efficacy of mammographic screening in reducing mortality from breast cancer is sufficient for women 50 to 69 years; efficacy for women in other age groups is considered inadequate” [6]. Together, these findings seem to echo Gøtzsche and Jørgensen’s 2013 Cochrane review, which states that the trade-offs of screening – such as over-diagnosis, over-treatment, and psychological distress – should be carefully considered in the decision to attend or support screening programs among all age groups [7].

Currently, many countries in Western Europe and North America offer organized breast cancer screening programs. Reflecting the lack of scientific consensus about screening efficacy, the precise recommendations pertaining to the age-thresholds for program inclusion vary. In the United States, the Preventive Services Task Force advises women between the ages of 50 and 74 to undergo a biennial screening mammography [8], while the National Breast Cancer Early Detection Program establishes an eligibility baseline for breast cancer screening for women aged 40-64. The American Cancer Society recommends yearly mammograms from the age of 45. In Europe, the EU Council advocates early detection services to be offered on a two-year basis to women aged 50 to 69 [9], but countries may implement these recommendations as they see fit. While some countries, like Norway, adhere to the thresholds as advised by the EU Council [10], other countries have implemented organized screening programs with more lenient age-thresholds. For instance, in the Netherlands all women between the ages of 50 and 75 are invited to participate in a biennial, nationwide screening program [11]. In Switzerland, programs are organized regionally. At present, eleven out of the twenty-six cantons offer screening programs to women over 50, with the age-limit varying between 69 and 74 [12].

Recent studies suggest that, in the face of conflicting research evidence and diverging recommendations, young women (30-49) often do not accept the lower age-thresholds for screening and advocate their right to be included in organized screening programs [13–15]. In fact, many young women already engage in opportunistic screening. Data from the U.S. National Health Interview Survey show that 29% of women aged 30-39 report to have had a mammogram [16]. Moreover, data from the 2010 Behavioral Risk Factor Surveillance Study reveal that 83% of women aged 40-49 have undergone mammography screening [17]. In the Netherlands, 37% of women aged 16 and over have had a mammogram in the past two years [18]. The Swiss Federal Statistical Office reports that 44% of women aged 40-49 have undergone at least one mammogram, almost half of them in the last two years [19]. Many of these women are unlikely to exhibit symptoms and use mammography as a mere precautionary measure.

There are several possible explanations for young women to seek mammographic screening against official recommendations and in absence of medical indications. First, women may not be aware of the lower age-thresholds. Breast cancer (screening) knowledge has been shown to be one of the key predictors of screening intentions among older women [20–23]. Second, ample media coverage of the debate about breast cancer and detection programs may in part induce erroneous beliefs about screening and arouse anxiety – particularly among women aged 40-49. For instance, Pink Ribbon International, one of the most visible non-profit organizations dedicated to raising breast cancer awareness and funding, explicitly advocates young women to have a screening mammogram: Triennially from the age of 20 and biennially from the age of 40 onward [24]. Likewise, public health efforts directed at convincing older women to engage in mammographic screening may – as an accidental byproduct – have motivated younger women to go for screening. Induced by fear of breast cancer and dying, an overestimation of their personal breast cancer risk, and overconfidence in the potential of mammographic screening, young women consequently seem to have adopted a perhaps overly positive attitude towards early detection practices [25]. Schulz and Meuffels [13–15] refer to such positive attitude based on perceived personal relevance as high ego-involvement with mammography screening.

To date, relatively little research has been done to investigate the precise motivations underlying young women’s intentions to go for mammographic screening prior to program eligibility [25]. Yet, such research is crucial when striving, on the one hand, to enforce policy decisions concerning the age-thresholds for systematic screening programs and, on the other hand, to allow young women to make evidence-based, informed decisions about mammography, both now and in the future. The present study, therefore, seeks to create a better understanding of young women’s motivations to engage in early detection practices. Building on the findings of prior studies, it reports on a survey that was conducted across Switzerland, focused on young women’s knowledge, fear, risk perceptions, and ego-involvement as possible drivers to engage in medically unwarranted mammographic screening before the age of 50.

## Methods

### Participants

In January 2015, a cross-sectional survey was conducted across all Swiss cantons among a sample of 918 Swiss female residents (i.e., in possession of a passport or residency permit), aged 30 to 49. Participants were recruited in collaboration with Polyquest, a Swiss-based market research agency that uses participant panels. The response rate was relatively low (16.5%) and the drop-out rate was high (26.25%). This was assumed to be due to survey length and sensitivity of the topic. Moreover, it was not allowed for participants to skip questions. While it was not possible to access personal data of those who had interrupted the survey, the sample ensured to provide a reflection of this particular segment of the Swiss population in terms of socio-demographics, including age, marital status, education, occupational status, and geographical location. For analytical purposes, the French- and Italian-speaking regions of Switzerland were oversampled relative to the German-speaking region. The overall sample size was determined on the basis of power calculations.

Prior to filling out the survey, participants were informed that the study was aimed at improving the understanding of women’s views on and concerns about breast cancer and organized mammographic screening programs. Moreover, they were notified that the study had been funded by the Swiss National Science Foundation and that the protocol had been reviewed and, where applicable, approved by the relevant ethical committees (Swiss Ethics Committee for research involving humans; Institutional Research Board of the University of Lugano). As approved by the ethics committees and following standard practice of the surveying partner, all participants provided informed consent within the online surveying tool and selected their preferred surveying language (French, German, or Italian) by clicking ‘next’. The survey took approximately 30 min to complete and had to be filled out in one sitting.

### Survey development

The survey consisted of standardized socio-demographic questions and validated scales. When necessary, the measures were adapted or newly developed, e.g., to account for the Swiss breast cancer (screening) context. All measures were translated from English to German, French, and Italian using a back-translation procedure. A pre-test (*n* = 22) confirmed the comprehensibility, accuracy, and feasibility of the online questionnaire.

#### Breast cancer (screening) knowledge

To measure participants’ breast cancer knowledge, the Comprehensive Breast Cancer Knowledge Test was used [26, 27]. To match the purpose and context of the study, the tool was adapted in several ways: items pertaining to breast self-examination were removed;, all items were reformulated into true/false questions and updated to the contemporary Swiss context, and, lastly five items concerning the organized *Swiss screening program* were added.

Knowledge scores were computed for each of the subscales and were expressed as the percentage of correctly answered questions. Biserial correlations of each separate item with the total score of the subscale were computed to determine the unidimensionality of each subscale. Items belonging to the *general knowledge, curability,* and *Swiss screening program* subscales all had biserial correlations between .21 and .57 and were significant at the *p ≤* .01 level. These items were retained, resulting. in a total pool of 25 binary items. To establish the internal consistency reliability for the three remaining subscales, the Kuder Richardson 20 (KR20) statistic was used. This statistic provides an index of test reliability for scales consisting of dichotomous items. The internal consistency of the overall 25-item scale was low, with KR20 = .36 (general knowledge, KR20 = .26; curability KR20 = .46; Swiss program KR20 = .21).

#### Breast cancer fear

Breast cancer fear was assessed using the validated instrument developed by Champion and colleagues [28]. This measure consists of eight items, using a 5-point Likert scale ranging from ‘strongly disagree’ to ‘strongly agree,’ and taps into dimensions of fear such as anxiety, depression, and uneasiness. Exploratory factor analysis using principal axis factoring confirmed the validity of the translated scale (KMO = .85), showing one factor, explaining 67.6% of the variance. Furthermore, the translated scale had high internal consistency with a Cronbach’s alpha of .94.. As such, these results demonstrated the validity of the scale translation.

#### Risk perception

Participants’ risk estimates were measured in two ways. Following Daly and colleagues [29], subjective risk perception was measured by asking participants to rate their chance of getting breast cancer someday on a scale from 0% (no chance) to 100% (definitely will get it). Additionally, a measure of perceived susceptibility to get breast cancer was added: a 5-item, 5-point Likert scale developed by Champion [31, 32]. Exploratory factor analysis with principal axis factoring (KMO = .85) confirmed one factor, explaining 62.3% of the variance. Cronbach’s alpha was .88.

#### Mammography screening involvement

Young women’s involvement with breast cancer screening practices was assessed using an adaptation of Zaichkowsky’s personal involvement inventory – an attitude measure based on semantic differentials [33]. While Zaichkowsky’s scale was originally developed to be used in the context of product purchases and advertising, it adopts a general view of involvement that focuses on personal relevance that is also largely applicable to breast cancer screening practices. To ensure an optimal contextual fit as well as to shorten the measure, similarly to Zaichkowsky’s revised personal involvement inventory [34], only half of the scale items were used. An exploratory factor analysis was performed using principal axis factoring (KMO = .96). The analysis confirmed one factor, explaining 62.8% of the variance. The scale demonstrated high internal consistency with a Cronbach’s alpha of .94.

#### Mammography intentions

Participants’ intention to have a mammogram was measured in two ways. First, participants were asked to respond to the statement “I intend to have (another) screening mammogram to check for breast cancer in the near future”, using a 5-point Likert scale (strongly disagree - strongly agree), adding the option ‘I have not yet thought about this’. Unless participants chose this last option, they were additionally asked at what age - provided that their health status would remain the same – they would consider to have a (next) mammogram.

### Data analysis

Data were analyzed using the statistical package SPSS (IBM, version 21). To test the associations between the variables of interest, Pearson’s correlation coefficient was used. Hierarchical multiple linear regression was performed to adjust for potential confounders and to order the importance of predictor variables. Variables were entered block-wise and in hierarchical order, starting with socio-demographic (control) variables (block 1), then adding the presumed predictors in a step-by-step procedure based on theoretical assumptions of causality and importance: perceived risk and susceptibility (block 2), perceived fear (block 3), and ego-involvement and knowledge (block 4). The results of the regression are reported with 95% confidence intervals and *p-*values. A *p-*value of < .05 was considered as statistically significant.

## Results

### Sample

Participants were on average 39.37 years old (*Md =* 39, *Mo =* 36, *SD =* 5.88, range: 30-49). 82.4% of participants indicated to have the Swiss nationality. Overall, 59.9% of participants hailed from the German-speaking region of Switzerland, 29.8% from the French-speaking region, and 10.2% from the Italian-speaking region. These numbers were reflected by participants’ choice of survey language (German: 60.1%; French: 29.8%; Italian: 10%). Cantons Zurich (14.1%), Berne (11.2%), and Vaud (11.1%) were most represented. In terms of screening availability, 52.18% of the participants lived in a canton which offered a systematic screening program at the time of the survey, while 47.82% lived in a canton offering opportunistic screening.

Most participants were married or in a stable relationship (63.8%). 21.7% of the participants were single, 14.5% divorced, separated, or widowed. With regard to their educational background, 8.3% of the participants indicated to have a middle school degree, 62% a professional or high school degree, 15.4% a degree from a university of applied sciences, and 14.2% a degree from a (polytechnic) university. Of all participants, 0.2% indicated not holding any degree. The majority of participants were (self)employed (57.6%). 25.4% of participants were homemakers, 8.8% were unemployed (of which 7.6% in search of employment), 0.7% were students, and finally 0.4% were pensioners.

Participants evaluated their overall health as relatively good: On a scale ranging from 1 (“very poor”) to 5 (“very good”), participants scored an average of 3.74 (*SD =* .88, range: 1-5). As expected, having been diagnosed with a chronic disease significantly impacted self-perceived health (F(1, 892) = 129.39, *p ≤* .001, η^2^ = .13). Furthermore, 27% of all participants indicated to have had a mammogram in the past. Lastly, 58 participants had been previously diagnosed with breast cancer (2.8%) or with a genetic predisposition to get this disease (3.5%). These participants were removed from the sample for subsequent analyses. See Table 1 for an overview of the sample description.

**Table 1:** Sample description

Participants (*N =* 918)	Number	Percent	M	SD	Range
Age	–	–	39.37	5.88	30-49
Marital status			–	–	–
Married/stable relationship	586	63.8			
Single	199	21.7			
Divorced/separated/widowed	133	14.5			
Education			–	–	–
None	2	.02			
Middle school	76	8.3			
Professional or high school	569	62			
University of applied sciences	141	15.4			
University	130	14.1			
Occupation			–	–	–
(Self)employed	594	57.6			
Homemaker	233	25.4			
Unemployed	81	8.8			
Student	6	.7			
Pensioner	4	.4			
Swiss nationality			–	–	–
Yes	756	82.4			
No	162	17.6			
Swiss language region			–	–	–
Swiss-German	550	59.9			
Swiss-French	274	29.8			
Swiss-Italian	94	10.2			
Systematic screening program			–	–	–
Yes	479	52.2			
No	439	47.8			
Health status	–	–	3.74	.88	1-5
Genetic predisposition (e.g., BRCA1)	32	3.5	–	–	–
Breast cancer diagnosis	26	2.8			
Past mammogram (*N =* 860)	232	27	–	–	–
30-34	23	10.1			
35-39	48	22.1			
40-44	59	29.4			
45-49	102	47.7			

### Descriptive statistics

Participants were relatively knowledgeable about breast cancer and mammography screening. On average, women answered 70.8% of the knowledge questions correctly, (*Md* = 72, *Mo =* 72, *SD =* 10.48), with a range between 28% and 96% corresponding to a minimum of seven and a maximum of 24 correct answers. In terms of *general knowledge*, women answered on average 66.9% of the questions correctly (*Md* = 66.7, *Mo =* 67, *SD =* 14.42, range: 25%-100%). The scores for *curability knowledge* were higher, with an average score of 78.1% (*Md* = 75.0, *Mo =* 88, *SD =* 17.75, range: 0%-100%). Lastly, with regard to *Swiss program knowledge*, women scored on average 68.2% (*Md* = 80.0, *Mo =* 80, *SD =* 21.26, range: 0%-100%). Close examination of the individual items revealed that while participants performed well on most questions, notably some questions elicited more erroneous than correct responses. These questions pertained in particular to the risk factors for developing breast cancer. Overall, participants displayed a lack of knowledge of known risk factors, such as being overweight, living in a Western country, and age. More specifically: 54.1% of participants did not know that being overweight is a risk factor for breast cancer; 56.3% of women were not aware that breast cancer is more common in Switzerland than in Africa and Asia. Moreover, 52.9% of participants answered (erroneously) that women without known risk factors rarely develop breast cancer. Further, 57.3% did not know that breast cancer is more prevalent under women aged 65 than 40. This latter finding was emphasized by the result that 42.1% of participants thought that women over 70 rarely develop breast cancer. In addition, 57.6% of women stated that mammograms are pain free. Lastly, participants were unaware of the age-thresholds for organized screening in Switzerland: The majority of participants (64.1%) answered that Swiss programs invite women from the age of 40 onward.

At the same time, participants appeared fearful of breast cancer and over-estimated their risk of getting breast cancer. On average, participants scored a 23.41 on the breast cancer fear scale, with scores ranging between eight and 40 (*Md* = 24.0, *Mo =* 24, *SD =* 7.64). Overall, 13.7% of the participants were characterized by low (score: ≤ 15), 30.2% by moderate (score: 16-23), and 56% by high levels of fear (≥24). When asked about their personal risk of developing breast cancer, participants on average estimated this at 20.45% (*Md* = 20.0, *Mo =* 10, *SD =* 19.06, range: 0-100), equivalent to a chance of 1 in 4.89. Participants’ self-perceived susceptibility to get breast cancer, however, was relatively low with a mean score of 2.24 (*Md* = 2.20, *Mo =* 1, *SD =* .82, range: 1-5).

Participants also appeared to be highly involved with the topic of breast cancer screening (*M =* 5.40, *Md* = 5.5, *Mo =* 7, *SD =* 1.12, range: 1-7). This implies that these women deem mammography screening of very high relevance to them personally and thus seem to have a very positive attitude towards mammography screening – despite their young age.

Finally, when asked to respond to the statement “I intend to go for mammography screening in the near future”, 87 participants (*N* = 860) indicated to not have thought about that yet. Among those who responded to the statement, 44.2% indicated to (strongly) disagree, 21.2% to neither agree nor disagree; and 34.5% to (strongly) agree. On average, these respondents scored a 2.90 (*Md* = 3.0, *Mo =* 2, *SD =* 1.33, range: 1-5). When asked at what age they intend to have their (next) screening mammogram (*N* = 773), 272 women (35.2%) answered they do not plan to have a mammogram at all. The remaining women, indicated an average age for a (next) mammogram of 45.50, with a mode of 50 (*Md* = 45.0, *SD =* 5.62, range: 31-80). In total, 56.7% of respondents indicated the intention to have (another) screening mammogram below the age of 50, thus below the lower age threshold for systematic screening in Switzerland (9.6% indicated an age below 40; 24.4% said 40-44; 22.8% answered 45-49). In the Swiss-Italian region the indicated average age was 44.10 (*Md* = 44.5, *Mo =* 40, *SD =* 6.89, range: 32-80), in the Swiss-German region 45.62 (*Md* = 46.0, *Mo =* 50, *SD =* 5.75, range: 32-60), and in the Swiss-French region 45.84 (*Md* = 46.5, *Mo =* 50, *SD =* 4.85, range: 31-55).

### Relationships between knowledge, attitudes, risk perceptions, and intentions

In a first step, the strength of the relationships between the variables of interest was tested. No meaningful associations were found between breast cancer knowledge and any of the other variables, including the main outcome variable ‘mammography intentions’. This applied to overall knowledge scores as well as to the three subscales. Bivariate correlation analyses revealed a significant, weak relationship between young women’s fear of breast cancer and their risk perceptions, as such that higher risk perceptions were associated with higher levels of fear (*r*(858) *=* .24, *p* ≤ .001). Perceived susceptibility to get breast cancer, as well, was strongly correlated with breast cancer risk perceptions and fear (*r*(858) *=* .54, *p* ≤ .001); (*r*(858) *=* .49, *p* ≤ .001), respectively). Breast cancer fear and susceptibility, in turn, were moderately correlated with participants’ ego-involvement with breast cancer screening (*r*(858) *=* .32, *p* ≤ .001); (*r*(858) *=* .30, *p* ≤ .001). That is, participants who perceived themselves as fearful and highly susceptible to breast cancer judged mammography as more relevant to them personally and, thus, had a more positive attitude towards screening. Finally, the intention to go for screening in the near future was strongly associated with participants’ ego-involvement (r(771) = .45, *p* ≤ .001), their perceived susceptibility (r(771) = .29, *p* ≤ .001), and fear (r(771) = .29, *p* ≤ .001). The results show an association between participants’ involvement, fear, and perceived susceptibilityand their intention to engage in screening in the near future. In Table 2, an overview of the associations between the different variables can be found.

**Table 2:** Pearson’s correlation coefficient for the variables studied

	Knowledge	Fear	Perceived risk	Perceived susceptibility	Ego-involvement
Knowledge	–				
Fear	-.02	–			
Perceived risk	.01	.24^***^	–		
Perceived susceptibility	.04	.49^***^	.54^***^	–	
Ego-involvement	.07	.32^***^	.19	.30^***^	–
Mammography intention	.02	.29^***^	.19	.29^***^	.45^***^

*Note.* ^***^Correlation significant at *p* ≤ 0.001. Only correlations above .20 have been considered for significance

Subsequently, a hierarchical multiple linear regression analysis was used to test if knowledge, perceived breast cancer risk and susceptibility, breast cancer fear, and ego-involvement can indeed predict participants' intentions to go for mammography screening. Participants’ age, educational levels, and geographical location were controlled for as possible confounder variables. The results of the regression analysis demonstrate that three main variables of interest, as well as two control variables, significantly predict mammography intention, namely: ego-involvement (*β* = .34, *p* ≤ .001), breast cancer fear (*β* = .08, *p* ≤ .05), perceived susceptibility (*β* = .10, *p* ≤ .05), geographical location (Swiss-French group: *β* = .15, *p* ≤ .001; Swiss-Italian group: *β* = .26, *p* ≤ .001), and age (*β* = .11, *p* ≤ .001). Together, these variables explain 32% of the variance (*R* ^*2*^ = .32, F(9, 772) = 38.78, *p* ≤ .001). Breast cancer knowledge, risk perceptions, and educational status did not significantly contribute to the final model (see Table 3).

**Table 3:** Coefficients variables resulting from hierarchical multiple linear regression analysis (final model)

Model variables	B	SE	β	*t*	*p*	R	R^2^	Adj. R^2^
Constant	-1.27	.45	–	-2.85	.008	–	–	–
Covariates								
Age	.025	.01	.11^***^	3.69	.001	–	–	–
Education	.075	.05	.05	1.53	.174	–	–	–
Region						–	–	–
Swiss-Italian	1.12	.14	.26^***^	8.14	.000			
Swiss-French	.43	.09	.15^***^	4.65	.000			
Predictors								
Ego-involvement	.40	.04	.34^***^	10.2	.000	–	–	–
Knowledge	-.27	.39	-.02	-.71	.389	–	–	–
Fear	.02	.01	.08^*^	2.34	.013	–	–	–
Susceptibility	.15	.06	.10^*^	2.36	.015	–	–	–
Risk	.00	.00	.03	.94	.388	–	–	–
Model	–	–	–	–	–	.56	.32	.31

*Note. B* unstandardized regression coefficient, *SE* standard error, *β* standardized regression coefficient, *t* obtained t-value, *p* probability, *R2* proportion variance explained, *Adj. R2* adjusted proportion variance explained. Dependent variable: mammography intentions in the near future. ^*^Correlation significant at *p*  ≤ 0.05. ^**^Correlation significant at *p*  ≤ 0.01. ^***^Correlation significant at *p* ≤ 0.001

## Discussion

The results of the present study shed light on what motivates young healthy women to engage in premature opportunistic breast cancer screening. Some of the main known predictors of breast cancer screening behaviors among women who are eligible for screening were tested for their explanatory potential among a young age group. These predictors are: breast cancer knowledge, breast cancer fear, perceived risk, and mammography involvement – or attitude. In summary, it can be said that, despite their relatively high levels of knowledge about breast cancer, the young women in our sample display high levels of breast cancer fear and tend to overestimate their risk of getting this disease, even though the objective risk within this particular age group without respective genetic predispositions is low. Also, young women’s ego-involvement with mammography screening is high. That is, participants judge mammography screening to be of high personal relevance and, thus, have a positive attitude towards screening – in spite of their young age. Even though overall screening intentions are moderate, the majority of participants plan to go for opportunistic screening before the age of 50.

This study shows that breast cancer screening knowledge is neither associated with nor predictive of screening intentions among young women. This is remarkable, because knowledge has been shown to be a good predictor of screening behaviors among women who are eligible for screening. [20, 21, 23]. Breast cancer fear, perceived susceptibility, and mammography involvement, however, do predict screening intentions – as do age and geographical location. Notably, Champion demonstrates that while moderate fear can predict screening behaviors, very high levels of fear can, in fact, prevent eligible women from engaging in organized screening [28]. Among the young age group, however, excess levels of fear seem to cause early detection practices.

Remarkably, given their overall good knowledge about breast cancer, women display a lack of knowledge of personal risk factors such as being overweight, older age, and genetic predisposition. Moreover, they believe that breast cancer is more common in Asia and Africa than close to home in Switzerland. Simultaneously, young women believe that mammograms are generally pain-free and systematically used in Switzerland to detect breast cancer among women aged 40 and over. On the one hand, these results point to an optimistic bias pertaining to the role of young Swiss women’s risk factors – some of which are within control, such as weight. On the other hand, the findings suggest that young Swiss women overestimate the medical potential of organized mammography screening. This may in part be caused by what has been termed the popularity paradox of screening: The more women are over-diagnosed and over-treated as a result of systematic early detection practices, the stronger the general belief that screening is effective in saving lives and the more popular screening programs become [35]. This is strengthened by messages in the media about improved survival statistics, which may in reality be an artefact of over-diagnosis rather than reduced mortality [36].

Taken together, the findings suggest that young women’s breast cancer screening behaviors are mostly motivated by emotional rather than rational factors. An important question that remains is what precisely causes young women’s elevated fear of breast cancer, their perceived susceptibility to breast cancer, and their involvement with mammography. Further studies, using more sophisticated statistical modeling techniques, could explore the factors which influence young women’s breast cancer fear and mammography involvement. Also, the influence of the media on young women’s perceptions and beliefs should be carefully examined. In addition, the role of the health care providers in influencing women’s decisions about mammographic screening needs to be closely considered. Indeed, recent research involving Australian breast screening experts, including clinicians as well as researchers, shows that there is still a lack of consensus on the experts’ side when it comes to communication about and framing of over-diagnosis as well as to the decision about how strongly women’s choices should be guided by an expert [37, 38]. This disagreement might be due to a different prioritization of ethical and epistemological values that, in addition to medical evidence, can impact the experts’ opinions about mammographic screening [37, 39].

Moreover, some of the present study’s findings warrant further exploration of the impact of culture, policy-making, and system factors on premature breast cancer screening behaviors. Descriptive statistics showed that Swiss-German participants’ are less likely to plan screening in the near future than Swiss-French and, particularly, Swiss-Italian participants. This may be because of cultural differences, as well regional differences in the implementation of organized screening. These regional variations were deemed beyond the scope of the present study, yet of considerable importance for national public health efforts. Therefore, a follow-up analysis has been dedicated to exploring the impact of cultural affiliation and program availability on screening perceptions and practices across the Swiss cantons more thoroughly.

While the results of the present study form a solid basis for further investigation, some limitations should be considered. First, participants of this cross-sectional survey were all members of Swiss survey panels and, as a result, may not be strictly representative of the population. This is a clear limitation. Moreover, in light of the relatively low response rate and high drop-out rate, it would have been particularly useful to compare those participants that completed the survey to those that did not. Second, the Comprehensive Breast Cancer Knowledge scale displayed unfavorably low internal consistency. Various authors have used (adaptations of) the Comprehensive Breast Cancer Knowledge Test and have reported varying KR20 results [40, 41]. It is possible that the scale adaptation and translation procedure have affected the low internal consistency of the scale and, in turn, the study’s results. Items that were relevant in the context of the original study may have been, e.g., less applicable to Swiss participants. Generally, it could be questioned whether a measure of internal consistency is useful to assess the reliability of a knowledge scale. After all, low internal consistency does not necessarily imply low reliability. While the scale findings thus have to be interpreted with some caution, they were nonetheless considered useful. When validating the present study, it would be recommended to use additional measures of breast cancer knowledge. Third, it was found that women’s risk perceptions did not significantly predict screening intentions and that, on average, women overestimated their risk. This could imply that women have difficulty to assess their risk numerically and that other measurement types should be preferred [30]. Fourth, as this is one of the first studies to quantitatively explore the factors underlying young women’s intentions to go for pre-mature screening, it focused on known predictors of mammography intentions among older women. However other variables could also be considered in future studies. Lastly, and perhaps most importantly, it should be kept in mind that the present study does not consider the legitimacy of women’s intentions. That is, as this study focused on healthy young women’s motivations, participants’ individual risk to develop breast cancer in the future, as measured by a breast cancer risk assessment tool [29], was not considered in the present study. Even though all participants who had been formally diagnosed with (a genetic predisposition to develop) breast cancer were excluded from this study, some women may have indicated a strong intention to go for screening in the near future for medically valid reasons (e.g., a recent medical exam or their family history).

## Conclusion

While ample studies have been dedicated to identifying the factors underlying women’s intentions to engage in breast cancer screening, these studies have predominantly focused on those women who are indeed eligible for screening. Instead, this study investigates what motivates young women’s premature opportunistic screening behaviors among a sample of Swiss participants aged 30 to 49. This target group is of high relevance for several reasons. As these women form the potential screening population of the future, it is helpful to understand what motivates their behavior prior to inclusion in systematic screening programs. After all, these women are not only exposed to extensive media coverage about breast cancer (screening), but also to campaigns promoting mammography screening among the actual target group. As such, their opinions may be shaped by these campaigns and, potentially and partially, be induced by fear. Data shows that young women engage in opportunistic screening relatively often and, moreover, are of the opinion that they should be included in organized screening programs [13–15, 17, 19]. The influence of healthcare providers on young patients’ decisions and options about this has thus far remained unclear. Yet, mammography overuse can have serious consequences. In light of the broad scientific, political, and public discussion about the efficacy of screening, it is therefore of pivotal importance that studies consider all age groups.

The present study demonstrates that young women are indeed inherently different from women who are eligible for screening in terms of what drives their mammography behaviors. Among young women, breast cancer fear, perceived susceptibility, and mammography involvement were found to be the main predictors of screening intentions. Breast cancer knowledge was not found to be predictive of their intention to engage in mammography. Additionally, young women did not always correctly identify breast cancer risk factors. Taken together, this implies that in order to successfully promote adherence to screening guidelines – whether these are based on unequivocal scientific evidence or policy decisions – different public health communication strategies must be developed for women of different age groups. In doing so, efforts should be directed towards informing young women about the correct age-thresholds for mammography, correcting their erroneous beliefs about breast cancer and screening, and reducing excess levels of fear and involvement. Meanwhile, attention should be paid that women are not necessarily discouraged to engage in organized screening once they have reached program eligibility. To do so, public health efforts should be supported by patients’ interpersonal exchanges with their own providers. Overall, the findings suggest that, rather than a rational and knowledge-oriented approach, a targeted approach that takes young women’s underlying motivational orientation and psychological distance into account, could be promising when designing interventions to justify and promote public health decisions concerning the age-thresholds for mammography. Moreover, effective communication interventions will allow young women to make better-informed decisions about screening, both now and in the future.
